# Physical Activity, Self-Care, and Menopausal Symptoms among Women in Al-Ahsa, Saudi Arabia: Adherence to Postmenopausal Guidelines (PMGs)

**DOI:** 10.3390/healthcare12090886

**Published:** 2024-04-24

**Authors:** Sahbanathul Missiriya Jalal

**Affiliations:** Department of Nursing, College of Applied Medical Sciences, King Faisal University, Al-Ahsa 31982, Saudi Arabia; sjalal@kfu.edu.sa; Tel.: +966-5640-70973

**Keywords:** menopausal symptoms, physical activity, self-care, post-menopausal guidelines, women, adherence

## Abstract

Menopause is a physiological change in which the menstrual period permanently ends. Every woman experiences this transition in different ways between the ages of 40 and 55. Women may have menopausal symptoms as a result of low estrogen levels. Self-care is a practice which women can use to maintain their wellness. This study aimed to assess physical activity, self-care, and menopausal symptoms and their associations with selected variables. The results showed that women should adhere to postmenopausal guidelines (PMGs). A cross-sectional study was conducted among 212 menopausal women randomly selected from health centers in Al-Ahsa, Saudi Arabia. The international physical activity tool, a self-care questionnaire, and the Kupperman menopausal index scale were used to assess women’s physical activity, self-care, and menopausal symptoms, respectively. The chi-square and Pearson correlation tests were used for analysis. The women were 55.01 ± 6.87 years old, and 40.6% reached menopause between the ages of 46 and 50 years; 57.1% of the women had low physical activity, which was associated with menopausal symptoms (*p* < 0.022). The highest mean score (29.63 ± 5.83) was obtained for physical health, while the lowest (11.92 ± 3.58) was found in self-care screening tests. Regarding menopausal symptoms, 25.9% had mild, 69.8% had moderate, and 4.3% had severe symptoms. A significant association was found between menopausal symptoms and age, menopausal age, education, body mass index, and PMGs awareness at *p* < 0.001. Self-care positively correlated with menopausal symptoms (*p* < 0.001). Therefore, attention should be given to women’s adherence to PMGs so that they can enjoy healthier lives after menopause.

## 1. Introduction

Menopause is a natural physiological phenomenon in middle-aged women and is associated with the gradual decline of ovarian follicles and their hormones [1]. Because of a decline in estrogen hormone levels, during the perimenopausal period, women may experience reduced physical wellbeing accompanied by various symptoms related to menopause, including psychological, physical, sexual, and vasomotor complaints [2]. Globally, 1.5 million women go through the menopausal transition each year. This change frequently brings about challenging symptoms such as vasomotor symptoms, vaginal dryness, joint pain, muscle aches, decreased libido, depression, irritability, hot flashes, memory loss, nervousness, bladder issues, insomnia, fatigue, and malaise, with the most frequent symptoms being muscle and joint issues [3,4,5].

According to the 2022 Saudi census results, this country’s population stands at 32,175,224, out of whom 18.8 million are Saudis. Of these, 12.5 million are women, making up 39 percent of the total population [6]. In addition, most Saudi women reach menopause between 51 and 55 years of age [7,8]. Physical activity has numerous positive effects on both physical health and psychological wellbeing [9]. Engaging in regular physical activity among menopausal women lowers the risk of hot flashes, osteoporosis, fractures, cardiovascular disease, and obesity [10]. Regular physical activity also significantly improves mental health, preventing the development of anxiety and depressive disorders, night sweats, and insomnia; reducing the symptoms of anxiety and depression if they already exist; and elevating feelings of wellbeing [11]. A study report showed that women who are routinely active report significantly fewer somatic symptoms and less pain than sedentary women [12]. Similarly, the mean somatic symptom score was 28% lower among active women in a large cross-sectional survey of physical activity, BMI, and health-related quality of life in British women [13]. Furthermore, reductions in somatic symptoms were highly related to increases in physical activity over time in a longitudinal study involving 3300 Australian women [14]. Frequent exercise, which includes aerobic training, resistance training, stretching, and relaxation techniques, improves overall health, lowers the risk of chronic illnesses, keeps bones and cartilage stronger to prevent osteoporosis, and helps maintain a healthy weight without causing gradual weight gain [15,16,17].

Self-care is important for overall health, but it is critical for women, especially those going through menopause. The World Health Organization (WHO) encourages self-care throughout this stage of life [18]. According to the WHO, self-care is defined as the ability of individuals, families, and communities to promote health, prevent disease, maintain health, and cope with illness and disability with or without the support of a healthcare provider [19]. Women who take the right self-care steps during menopause can enhance their quality of life. The actions that people carry out on their own behalf to preserve life, health, and wellbeing are the central idea in self-care theory. Many theorists have emphasized the priority of self-care and demonstrated how crucial it is to advance and promoting health [20,21]. Therefore, it is necessary for women to follow the postmenopausal guidelines (PMGs), which are detailed by the Ministry of Health of Saudi Arabia on its website [22].

To the best of my knowledge, no research has been conducted on menopausal women’s practices regarding physical activity and self-care or their association with symptoms in Al-Ahsa, Saudi Arabia. As healthcare professionals, it is our responsibility to determine whether women adhere to postmenopausal guidelines (PMGs). Therefore, this study aims to assess the physical activity and self-care practices of menopausal women and to associate the selected variables with menopausal symptoms. This will help women follow PMGs and thus reduce morbidity after menopause.

## 2. Materials and Methods

### 2.1. Study Design 

A descriptive, cross-sectional research design was used to assess physical activity, self-care, menopausal symptoms, and their associations with variables among women after menopause. This cross-sectional study was conducted from September 2023 to December 2023 and followed STROBE (strengthening the reporting of observational studies in epidemiology) guidelines.

### 2.2. Study Area and Setting

This study was conducted among menopausal women to identify their use of physical activity and self-care associated with menopausal symptoms in selected primary health centers (PHCs) in Al-Ahsa, located in the eastern region of Saudi Arabia. Approximately 1.3 million people live in this area, with around 43% being female. About 70,000 women are above 40 years old. Most women visit PHCs for primary healthcare services and regular treatment of chronic illnesses. 

### 2.3. Sample Size and Sampling

Based on the study’s characteristics, results, and outcomes—and assuming 50% of the study population has menopausal symptoms, as with menopausal women in a previous study [23] in Riyadh, Saudi Arabia, with an acceptable margin of error of 6% at a 95% confidence level—a sample size of 266 was calculated using the formula *n* = 4pq/D2. After randomizing the sample, the researcher disseminated questionnaires to all women at the selected PHCs. Considering the inclusion criteria, 212 menopausal women were included in the data collection. According to the exclusion criteria, 54 women were not involved in this study, and 12 women refused to participate.

### 2.4. Inclusion and Exclusion Criteria

Participants were women aged 40 years and above who had reached menopause, experienced one or more menopausal symptoms, and were attending PHCs in Al-Ahsa, Saudi Arabia. Women who could read and understand Arabic or English were included in the data collection. The study comprised women who provided their informed consent for data collection and publication. Women with severe chronic diseases such as stroke, renal failure, multiple organ failure, bone fracture, lung diseases, breast cancer, ovarian tumor, or uterine issues were excluded from the study. Women who reached menopause because of surgery like hysterectomy, oophorectomy, or medically induced menopause, or who were on hormonal replacement therapy, were excluded from this study. 

### 2.5. Data Collection Tool and Procedure 

The data were collected using structured self-administered questionnaires to assess the physical activity, self-care, and menopausal symptoms of the women. There were four parts to this questionnaire. 

The first part included socio-demographic variables about the menopausal women. The second part focused on physical activity. The third part addressed the self-care practices of the women. The fourth part covered menopausal symptoms. The tool used to gather the data was initially developed in English and later translated into Arabic. Three doctors of gynecological nursing, three gynecologists, and three family physicians who are experts in clinical and healthcare research reviewed the structured questionnaire (both the English and Arabic versions) to assess its validity, clarity, and feasibility for data collection. Some modifications were made to the tool based on the experts’ suggestions. As adherence to PMGs was the focus of this study, the questionnaire was developed based on the recommendations for women’s reproductive health and self-care that were posted on reliable organizations’ websites, such as Women’s Preventive Services Initiatives (WPSI) 2021 recommendations for well-woman care [24], WHO women’s health [25], and PMGs recommended by the Ministry of Health of Saudi Arabia [22]. The survey tool included questions related to adherence to PMGs [26].

A pilot study was conducted among 15 menopausal women to test the tool. The reliability of the questionnaire was assessed (r = 0.89) using Cronbach’s alpha. The time taken to fill in the questionnaire ranged from 25 to 30 min. The study’s objectives were clearly explained to the participants before the start of data collection. The privacy of the respondents was ensured by not requesting personal identity information such as names or identity numbers in the questionnaire. All data were used for this study, and to maintain privacy and confidentiality, they were electronically encrypted and stored in a secure location with a password accessible only to the principal investigator. After obtaining written informed consent from each participant with clear and detailed information provided to them, a well-structured questionnaire was distributed to gather all the data in addition to the practice instrument.

#### 2.5.1. Demographic Variables

The demographic data parameters for the menopausal women included age, age at which women attained menopause, education level, occupation, marital status, nationality, number of children, body mass index (BMI) [27] (calculation shown in Table 1), awareness of PMGs, and adherence to PMG recommendations.

#### 2.5.2. Physical Activity Questionnaire

This instrument included the extended International Physical Activity Questionnaire (IPAQ) to evaluate degrees of physical activity in four areas: work, domestic chores, transportation, and leisure time, including walking and both vigorous and moderate activity during the previous week [28]. The results of the continuous score IPAQ were expressed as MET-min per week. MET is a measure of oxygen consumption during rest, or 3.5 mL O_2_/kg of body mass per minute. A high physical activity level is vigorous-intensity activity accumulating at least 1500 MET-min/week. Moderate physical activity includes achieving a minimum of at least 600 MET-min/week. Women who do not meet either category are considered to have low physical activity or to be inactive. 

#### 2.5.3. Self-Care Questionnaire 

The updated WPSI 2021 recommendations for women’s healthcare served as the basis for creating this questionnaire [24]. This structured tool has 38 items divided into four categories. The first category is physical health, which has 14 items. The second category is psychosocial health, which has 6 items. The third category includes reproductive–sexual health, which has 12 items. The fourth category is screening tests, which includes 6 items. A score of one was provided for the answer “No, I did not”. A score of two was provided for the answer “Yes, to some extent or I intend to do”. A score of three was provided for the answer “Yes, I did”. The domains with total score ranges available were physical health (14–42), psychosocial health (6–18), sexual–reproductive health (12–36), and screening tests (6–18). The final score range was between 34 and 190. The following equation was used to convert the total score and the scores for each domain into a standardized score between 0 and 100: (X − Min Score/Max − Min Score) [26]. 

#### 2.5.4. Menopausal Symptoms Questionnaire 

The Menopause Rating Scale (MRS) is used to assess menopausal symptoms. This standardized scale has been translated into many languages and is widely used to differentiate menopausal symptoms in women [29,30]. This self-administered instrument is filled out by women who have subjective complaints. The tool consists of 11 items categorized into three domains: the physical domain (four symptoms: sweating or hot flashes, cardiac complaints, sleeping disorders, and joint and muscle complaints); the psychological domain (four symptoms: depression, irritability, anxiousness, and exhaustion); and the urogenital domain (three symptoms: sexual problems, urinary problems, and vaginal dryness). Each symptom’s severity is rated on a scale from zero (none) to three (severe). The scores for each category are as follows: physical (0–12), psychological (0–12), and urogenital (0–9). A total score of 33 represents the maximum degree of complaints.

### 2.6. Ethical Considerations

Ethical approval was obtained from the Research Ethics Committee, Deanship of Scientific Research, King Faisal University, Al-Ahsa, Saudi Arabia (KFU-REC-2023-JUN-ETHICS969). Written informed consent was obtained from all participants before data collection. They were informed about confidentiality, the lack of risk, anonymity, and voluntary participation. The research protocol was approved by the institutional review board of the hospital in Hofuf, Saudi Arabia. After assessing the inclusion criteria, the study’s objectives and research purposes were clearly explained to all participants during data collection. They were permitted to withdraw from the study at any stage according to their interests. All participants were assured that their data would remain confidential. This study was conducted in accordance with the Declaration of Helsinki and followed ethical principles.

### 2.7. Statistical Analysis

Statistical Package for Social Sciences (SPSS) (IBM Corp. Released 2012. IBM SPSS Statistics for Windows, Version 21.0. Armonk, NY, USA: IBM Corp.) was used to analyze the study data. The numbers and percentages were tabulated in the form of frequency distribution, mean, and standard deviation calculated by using descriptive analysis. Chi-square analysis was used to test the association between menopausal symptoms with the selected demographic variables and physical activity. Pearson’s correlation test was used to determine the relationship between menopausal symptoms and self-care with a *p*-value (two-tailed) equal to 0.05 or less.

## 3. Results

### 3.1. Demographic Variables of Menopausal Women

A total of 212 menopausal women were included in the analysis (Table 1), of which 103 (48.6%) were in an age range of 51–60 years. The mean age of the women was 55.01 (SD ± 6.87) years. Approximately 86 (40.6%) reached menopause between the ages of 46 to 50 years, and 79 (37.3%) reached menopause at the age of 51 to 55 years. In terms of education, 95 (44.8%) had a primary level of education, and 56 (26.4%) studied up to high school or a higher secondary level. Regarding occupation, most women (135, 63.7%) were housewives, of which 178 (83.9%) were married. The majority (179; 84.4%) were Saudis, while the remaining 33 (15.6%) were non-Saudis. In total, 85 (40.1%) had four children or more. In terms of BMI status, 56 (26.4%) were normal and 54 (25.5%) were obese. Only 16 (7.5%) women were aware of PMGs, while the rest were unaware, with only 5 (31.3%) following these guidelines.

### 3.2. Physical Activity of Menopausal Women

The frequency distribution of menopausal women is shown in Table 1, with 121 (57.1%) engaging in a low level of physical activity, 67 (31.6%) engaging in a moderate level of physical activity, and 24 (11.3%) showing a high level of physical activity.

### 3.3. Self-Care of Menopausal Women

In Table 2, the descriptive statistics of self-care among menopausal women show that the mean scores and standard deviations for physical health, psychosocial health, reproductive health, and screening tests were 29.63 ± 5.83, 13.29 ± 3.65, 26.7 ± 6.75, and 11.92 ± 3.58, respectively. These results indicate that the highest mean score was in the physical health category of self-care, while the lowest was in screening tests. 

### 3.4. Menopausal Symptoms

The women showed mild, moderate, and high menopausal symptoms given on the MRS scoring shown in Table 1. Out of 212 women, 57 (25.9%) had mild symptoms, 138 (69.8%) had moderate symptoms, and the remaining 17 (4.3%) had severe symptoms. This indicates that none of them lacked menopausal symptoms. The mean score of symptoms in the different domains, physical, psychological, and urogenital, are provided in Table 3. In the physical domain, the highest mean score with SD was 1.55 ± 0.76 in joint and muscle complaints; in the psychological domain, the highest was 1.67 ± 0.82 in anxiety; and in the urogenital domain, the highest mean score was 1.94 ± 0.87 in suffering from vaginal dryness.

### 3.5. Associaton of Menopausal Symptoms with Demographic Variables 

There was a statistically significant association between menopausal symptoms and age (*p* < 0.001), age at menopause (*p* < 0.001), education (*p* < 0.001), nationality (*p* < 0.008), BMI (*p* < 0.001), and awareness of PMGs (*p* < 0.001). There was no significant association between menopausal symptoms and occupation, marital status, or number of children. Various levels of physical activity were associated with menopausal symptoms (*p* < 0.022), as shown in Table 4. The relationship between menopausal symptoms and self-care is presented in Table 5. A positive correlation was found between various domains of self-care and different categories of menopausal symptoms (*p* < 0.001), except for depression and irritability in the psychological domain.

## 4. Discussion

Menopause is an unavoidable biological process that occurs in middle-aged women. It marks the end of the menstrual cycle and is diagnosed after 12 months of the cessation of menstrual periods. Menopause can cause physical symptoms such as hot flashes, as well as emotional symptoms that can affect sleep, body weight, energy levels, and emotional health [31]. 

In the current study, women who reached menopause experienced mild, moderate, and high symptom levels, per the MRS results. Most of them had mild to moderate symptoms, while some had severe symptoms. A previous study analyzed the prevalence of menopausal symptoms and their associations with patient adherence. Most women reported symptoms at various levels, with hot flashes, sleep issues, bladder problems, vaginal dryness, and joint and muscle pain being common between 12 and 24 months of follow-up [32]. Another study observed subethnic differences in menopausal symptoms, with most women commonly reporting severe symptoms. The overall number of symptoms and their severity levels varied according to subethnicity in that study [33]. 

A previous study assessed vasomotor problems and other menopausal symptoms. At baseline, around one-third of the participants reported having at least one moderate-to-severe symptom. Age-related decreases in symptoms were observed, except for joint pain/stiffness, which remained consistent throughout the age groups [34]. The present research showed that the highest mean score could be observed in joint and muscle complaints under the physiological domain. One of the most common health issues among women going through menopause is poor sleep. The menopause transition can affect sleep at all phases. Another study showed that menopausal women are affected by poor sleep quality [35]. In the current study, the results also showed that most women have sleep problems. 

A cross-sectional study was performed in Mangalore to evaluate the patterns and intensity of menopausal symptoms and identify the variables linked to them [36]. In that study, the participants’ average age and the average age upon reaching menopause were similar to the present study. The most common symptoms were physical and mental issues such as joint and muscular soreness. Perimenopausal women reported higher rates of somatic and urogenital complaints, while postmenopausal women reported higher rates of somatic symptoms. A study was carried out in a rural area of West Bengal to assess the quality of life of perimenopausal women. The study showed that, of those who reported avoiding intercourse with a partner, half of them experienced overall sexual changes, and many reported vaginal dryness [37]. The present study showed that the most significant symptoms were anxiety in the psychological domain and vaginal dryness in the urogenital domain.

A previous investigation was conducted to determine the associations between self-reported physical activity and menopausal symptoms. This preliminary research found a stepwise correlation between moderate and intense physical activity and a reduced overall menopausal symptom score [38]. To ascertain the effects of body composition, fat-free mass index, and physical activity on total menopausal symptoms, a study was conducted on premenopausal, perimenopausal, and postmenopausal women. A significant association was found between body composition, physical activity, and menopausal symptoms [39]. In the current study, most women reported low physical activity, and there was also a significant association between physical activity and menopausal symptoms. Another study was conducted to determine whether promoting physical activity reduces menopausal symptoms. That research aimed to evaluate the efficacy of a behavioral approach for initiating and maintaining physical activity to minimize menopausal symptoms, and this proved successful [40].

A randomized-controlled study of a clinical experiment examined the impact of a self-care education program on postmenopausal women’s marital satisfaction and the severity of menopausal symptoms. A significant negative association was found between the degree of menopausal symptoms and marital satisfaction [41]. Another study was conducted to assess the impact of self-care education on menopausal women’s quality of life and self-care using the health literacy index. The results showed that the quality of life and self-care of menopausal women can be significantly enhanced by health-literacy-index-based self-care education, which can also be utilized to improve menopausal women’s health-related outcomes [42]. In the present study, a positive correlation was identified between the various domains of self-care and the different categories of menopausal symptoms.

The strength of this study is the use of a cross-sectional survey, which was randomized regarding the selection of participants, wherein the self-care of women was assessed and correlated with menopausal symptoms. However, this study also has some limitations. The potential confounding bias of the natural aging process on postmenopausal symptoms could not be ruled out. Furthermore, there is a chance of recall bias because the MRS relies on recall data. There is also a risk of introducing bias from self-reporting. The weakness of this study is that the quality of life of menopausal women [43] was not assessed. Also, no interventions were provided to decrease menopausal symptoms. Therefore, I recommend conducting a future experimental study on postmenopausal women to evaluate the effectiveness of PMGs. 

## 5. Conclusions

This study showed that menopause contributes to physical, mental, and urogenital health issues. Menopausal women should be educated; engage in adequate physical activity; practice good self-care; and be made aware of PMGs to enhance their quality of life on an individual and communal level. Nurses are responsible for promoting adherence to these guidelines to postmenopausal women to reduce mortality and morbidity rates and help middle-aged and elderly women lead healthier lives.

## Figures and Tables

**Table 1:** Frequency Distribution of Variables of Menopausal Women (*n* = 212).

Demographic Variables		No	(%)
Age (years)	40–50 years	66	31.1
	51–60 years	103	48.6
	More than 60 years	43	20.3
Age of menopause attainment	40–45	47	22.2
	46–50	86	40.6
	51–55	79	37.3
Education	Non-literate	29	13.7
	Primary level	95	44.8
	High or Higher Secondary school	56	26.4
	College & Others	32	15.1
Occupation	Housewife	135	63.7
	Self-employed	32	15.1
	Employed in Government	18	8.5
	Employed in Private	27	12.7
Marital Status	Married	178	83.9
	Divorced	12	5.7
	Separated	10	4.7
	Widow	12	5.7
Nationality	Saudi	179	84.4
	Non-Saudi	33	15.6
Number of Children	None	3	1.4
	1	18	8.5
	2	37	17.5
	3	69	32.5
	4 and above	85	40.1
BMI	Underweight < 18.5	14	6.6
	Normal weight = 18.5–24.9	56	26.4
	Overweight = 25–29.9	88	41.5
	Obesity ≥ 30	54	25.5
Aware of PMGs	Yes	16	7.5
	No	196	92.5

No—number; %—percentage; BMI—body mass index; PMGs—postmenopausal guidelines.

**Table 2:** Descriptive statistics of Self-care of Menopausal Women (*n* = 212).

Category	Number of Items	Range	Mean	SD	Skewness	Kurtosis
Physical health	14	14–42	29.63	5.83	0.31	2.33
Psychosocial health	6	6–18	13.29	3.65	−0.42	2.38
Reproductive-sexual health	12	12–36	26.7	6.75	−0.43	1.99
Screening tests	6	6–18	11.92	3.58	0.32	2.13

SD—Standard deviation.

**Table 3:** Mean Score of Menopausal Symptoms (*n* = 212).

Domain	Symptoms	Mean	SD	Variance *s*^2^
Physical	Sweating or hot flashes	1.1	0.91	0.82
	Cardiac complaints	1.0	0.75	0.56
	Sleeping disorders	1.16	0.66	0.44
	Joint and muscle complaints	1.55	0.76	0.57
Psychological	Depressed	0.77	0.60	0.37
	Irritable	1.4	0.77	0.59
	Anxious	1.67	0.82	0.67
	Exhausted	0.91	0.98	0.96
Urogenital	Sexual problems	1.70	0.82	0.68
	Urinary problems	1.36	0.78	0.61
	Vaginal dryness	1.94	0.87	0.76

SD—Standard deviation.

**Table 4:** Association of Menopausal Symptoms with Demographic Variables (*n* = 212).

Demographic Variables		Mild	Moderate	Severe	X^2^
Age (years)	40–50 years	35	28	3	X^2^ = 46.847 *p* < 0.001 *
	51–60 years	21	77	5	
	More than 60 years	1	33	9	
Age of menopause attainment	40–45	15	29	3	X^2^ = 24.35 *p* < 0.001 *
	46–50	34	50	2	
	51–55	8	59	12	
Education	Non-literate	7	16	6	X^2^ = 44.656 *p* < 0.001 *
	Primary level	15	71	9	
	High or Higher Secondary	13	42	1	
	College & Others	22	9	1	
Occupation	Housewife	38	81	16	X^2^ = 9.15 *p* < 0.1655 NS
	Self-employed	12	20	1	
	Employed in Government	8	9	1	
	Employed in Private	12	10	5	
Marital Status	Married	73	91	14	X^2^ = 8.88 *p* = 0.18 NS
	Divorced	4	7	1	
	Widow	1	8	1	
	Separated	1	10	1	
Nationality	Saudi	41	122	16	X^2^ = 9.65 *p* = 0.008 *
	Non-Saudi	16	16	1	
Number of Children	None	1	1	1	X^2^ = 14.003 *p* = 0.0817 NS
	1	8	8	2	
	2	14	21	2	
	3	15	51	3	
	4 and above	20	53	12	
BMI	Underweight < 18.5	11	2	1	X^2^ = 36.915 *p* < 0.001 *
	Normal weight = 18.5–24.9	16	39	1	
	Overweight = 25–29.9	20	64	4	
	Obesity ≥ 30	10	33	11	
Aware of PMG	Yes	13	2	1	X^2^ = 26.444 *p* < 0.001 *
	No	44	136	16	
Physical Activity	Low	23	86	12	X^2^ = 11.465 *p* < 0.022 *
	Moderate	23	39	05	
	High	11	13	0	

X^2^—Chi-square; * significant; NS—non-significant; *p* < 0.05.

**Table 5:** Relationship between Menopausal Symptoms and Self-care (*n* = 212).

Domain	Symptoms	R Value	*p* Value
Physical	Sweating or hot flashes	0.248	0.001 *
	Cardiac complaints	0.232	0.001 *
	Sleeping disorders	0.154	0.025 *
	Joint and muscle complaints	0.294	0.001 *
Psychological	Depressed	0.101	0.142 NS
	Irritable	0.088	0.2 NS
	Anxious	0.19	0.006 *
	Exhausted	0.294	0.001 *
Urogenital	Sexual problems	0.276	0.001 *
	Urinary problems	0.249	0.001 *
	Vaginal dryness	0.37	0.001 *
Overall		0.27	0.001 *

R—Pearson correlation; * significant; NS—non-significant; *p* < 0.05.

## Data Availability

Data are contained within the article.

## Abbreviations

BMI	Body mass index
IPAQ	International physical activity questionnaire
PHC	Primary health centre
MET	Metabolic equivalent task
MRS	Menopause rating scale
PMG	Post-menopausal guidelines
SD	Standard deviation
SPSS	Statistical Package for Social Sciences
STROBE	Strengthening the reporting of observational studies in epidemiology
WHO	World health organization
